# Paths of Aging Minds: Health Journeys of Older Adults with Mild Cognitive Impairment

**DOI:** 10.21203/rs.3.rs-11366061/v1

**Published:** 2026-11-01

**Authors:** Ning Zhang, Michelle Liu, Jinghong Gu

**Affiliations:** Northeastern University; Algonquin Regional High School; Northeastern University

**Keywords:** Mild cognitive impairment, dementia, Alzheimer’s disease and related dementias, cognitive trajectories, cognitive reversion, multistate Markov model, Health and Retirement Study

## Abstract

**Background:**

Mild cognitive impairment (MCI) is often conceptualized as an intermediate stage between normal cognition and dementia. However, emerging evidence suggests that cognitive status may change bidirectionally, with individuals experiencing reversion to normal cognition, persistent impairment, or progression to dementia. Previous studies have focused on single transitions or clinical samples frequently. This study aimed to characterize dynamic transitions among normal cognition, MCI, and dementia following incident MCI using longitudinal population-based data.

**Methods:**

We conducted a retrospective cohort study using data from the Health and Retirement Study (HRS) from 1992 to 2022. Cognitive status was classified at each biennial assessment using the Langa-Weir cognitive classification based on the modified TICS and proxy assessments. Incident MCI was defined as a transition from previously observed normal cognition to MCI. Participants were followed for up to five subsequent HRS waves (approximately 10 years). Descriptive statistics were used to describe changes of cognitive status between waves. A continuous-time multistate Markov model wase used to estimate transition intensities and transition probabilities among normal cognition, MCI, and dementia, including both progression and reversion.

**Results:**

Among 10,489 participants with incident MCI, the mean age at the incident MCI wave was 70.6 years (SD = 11.53), and 56.6% were female. Two years after incident MCI, 62.3% of observed participants were classified as having normal cognition, 31.2% remained in MCI, and 6.5% were classified as having dementia. At 4, 6, 8, and 10 years after incident MCI, the proportion classified as normal cognition declined while the proportion of participants with dementia increased. The multistate model demonstrated substantial bidirectional movement among cognitive states. The estimated transition intensity from MCI to normal cognition was 0.453 (95% CI 0.443–0.463), compared with 0.126 (95% CI 0.121–0.131) for MCI to dementia. Transitions also occurred from normal cognition to MCI (0.265; 95% CI 0.257–0.273) and dementia (0.037; 95% CI 0.035–0.041), as well as from dementia to MCI (0.226; 95% CI 0.209–0.245) and normal cognition (0.064; 95% CI 0.055–0.074).

**Conclusions:**

Cognitive trajectories following incident MCI were heterogeneous and dynamic, involving reversion, persistence, progression, and recurrent transitions among cognitive states. These findings support a multistate conceptualization of cognitive aging rather than a predominantly unidirectional model of progression toward dementia. Although reversion in cognitive classification should not be interpreted as definitive biological recovery, its frequent occurrence highlights the importance of repeated longitudinal assessment and further investigation of factors associated with cognitive resilience and dementia risk.

## Background

Alzheimer’s disease and related dementias (ADRD) represents a major and growing public health challenge worldwide. According to the World Health Organization, approximately 57 million people living with ADRD globally and the associated societal costs were estimated at US$1.3 trillion in 2019.(1) As populations age, understanding changes in cognition before the onset of dementia has become increasingly important for identifying opportunities to preserve cognitive function, and potentially delay progression to ADRD. Mild cognitive impairment (MCI) indicates a particularly important stage in the cognitive aging continuum because people with MCI continue to function independently in everyday life. (2)

MCI has usually been viewed as an intermediate or prodromal stage of dementia between normal cognition and dementia.(3) However, instead of a simple, unidirectional model of cognitive decline, growing evidence offered a more dynamic perspective of MCI: individuals with MCI may progress to dementia, remain cognitively impaired, revert to normal cognition, or die before another cognitive transition occurs. (4–6) A recent systematic review and meta-analysis of 89 studies found substantial differences in MCI prognosis across study settings. Dementia conversion was present in 41.5% of participants in clinical samples and 27.0% in population-based samples, whereas prevalence of reversion to normal cognition was 8.7% and 28.2% in these two samples, respectively.(5) Longitudinal studies further demonstrate that MCI is not necessarily a one-way transition toward dementia. Individuals may move from MCI back to normal cognition, and those who revert have been shown to have a substantially lower subsequent risk of dementia than individuals with persistent MCI, supporting the notion that cognitive status can improve as well as deteriorate over time. (7, 8) These findings suggest that cognitive aging may be better understood as a dynamic process of transitioning between cognitive states rather than as a fixed progression from normal cognition to MCI and ultimately dementia.(9)

However, several important knowledge gaps remain. First, much of the existing literature has focused on a single transition, particularly progression from MCI to dementia.(10–13) This may obscure the dynamic nature of cognitive change. Reversion from MCI to normal cognition, for example, does not necessarily represent a stable endpoint; individuals who revert may subsequently remain cognitively normal, return to MCI, or progress to dementia. Likewise, individuals in other cognitive states may experience subsequent improvement or deterioration. Thus, progression, persistence, and reversion of MCI are not isolated outcomes but interconnected components of a longitudinal cognitive trajectory.(9) Examining these simultaneous and repeated transitions may provide a more complete understanding of how cognitive status evolves over time. Another knowledge gap is that findings derived from clinical samples may not fully reflect cognitive transitions in the broader community-dwelling population. Population-based longitudinal data with repeated, consistently measured cognitive assessments are particularly valuable for distinguishing persistent cognitive states from recurrent changes over time.

To address these gaps, we examined cognitive changes following incident MCI by conceptualizing cognition as a dynamic multistate process including normal cognition, MCI and dementia, based on longitudinal HRS data. Rather than focusing exclusively on progression to dementia or reversion to normal cognition, we characterized bidirectional and recurrent transitions among all three cognitive states over approximately 10 years of follow-up. This approach provides a more comprehensive characterization of the heterogeneity of cognitive aging and the extent to which cognitive status may improve, persist, deteriorate, or change repeatedly over time.

## Methods

### Data Source and Study Design

This study is a retrospective cohort study of older adults based on data from the HRS from 1992 to 2022. The HRS is a longitudinal study that follows a nationally representative sample of US residents 50 years and older through biennial interviews. The survey collects information on demographic characteristics, health and functional status, employment, cognition, and physical function. Cognitive measures have been included in the HRS core survey since 1992. The HRS also incorporates proxy interviews for respondents who are unable to complete the interview themselves, an approach that helps reduce potential attrition bias related to cognitive impairment. This study used the RAND HRS Longitudinal File, which is a publicly available and streamlined dataset, to identify respondents with MCI onset, and to characterize subsequent changes in cognitive status.(14) This study was approved by the Northeastern University Institutional Review Board.

### Assessment of Incident MCI and Study Cohort

Cognitive status was determined at each available HRS interview.(15, 16) For self-respondents, cognitive functioning was measured using a 27-point scale derived from the modified Telephone Interview for Cognitive Status (TICS). The assessment includes immediate and delayed word recall, serial subtraction by 7, and backward counting, with higher scores indicating better cognition. Using the Langa-Weir classification, respondents were categorized into 3 mutually exclusive cognition states: normal cognition (12–27), MCI (7–11), or demented (0–6). These categories are research classifications of cognitive status rather than clinical diagnoses. If the respondents were unable to complete the cognitive assessment directly, cognitive status was determined using information obtained from proxy interviews. HRS reviewers collected a proxy assessment based on the proxy’s assessment of the respondent’s memory levels, the number of limits in instrumental activities of daily living, and the interviewer’s assessment of the respondent’s cognitive limit. Total proxy scores range from 0–9 points with higher scores indicating poorer cognitive performance. Scores of 5–9 indicate dementia, 3–4 indicate MCI, and 0-2- indicate normal cognition.

To establish incident cognitive impairment, respondents must have normal cognition followed by a subsequent qualified scores of MCI. The first wave at which a respondent transitioned from normal cognition to MCI was defined as the index MCI wave (baseline). Respondents without a prior observed normal cognitive state were excluded because incident onset of MCI could not be established. Beginning at the index MCI wave, respondents were followed for up to five subsequent HRS waves (every two years a wave), corresponding to approximately 10 years of follow-up. The final analytic cohort included 10,489 respondents who contributed at least one observed cognitive transition during follow-up.

### Main Outcome Measures

The primary outcomes of interest were transition probabilities between cognitive states, i.e., normal cognition, MCI, or dementia. Cognitive status was reassessed at baseline and each available biennial HRS wave throughout the follow up period. Because respondents could remain in their current cognitive state or transition to another state between successive waves, nine possible state-to-state patterns were considered: (1) MCI-normal state, (2) remain at MCI state, (3) MCI-dementia, (4) normal-MCI, (5) remain normal cognition, (6) normal-dementia, (7) dementia-normal, (8) dementia-MCI, and (9) remain dementia. Survey participants’ outcomes were followed up every wave. This framework allowed cognitive change to be characterized as a dynamic process that included both progression and reversion rather than solely as unidirectional progression toward dementia.

### Statistical Analysis

Baseline cognitive status and respondents’ characteristics of the study cohort were summarized using descriptive statistics.

The Multistate Markov model was used to characterize longitudinal transitions among normal cognition, MCI and dementia.(17) This method allows individuals to occupy one of several mutually exclusive states at a given time and to transition between states over subsequent observation periods. This approach is particularly useful for representing cognitive impairment as a dynamic process in which both deterioration and improvement may occur. In particular, all respondents in our study sample entered the multistate analysis in the MCI state because baseline was defined as the first observed transition from normal cognition to MCI. Cognitive state was subsequently assessed at each available follow-up wave. The model considered transitions among the three cognitive states throughout follow-up, including 9 possible states, as permitted by the specified transition structure. Remaining in the same cognitive state between successive waves was also incorporated into the estimation of state occupancy and transition probabilities.

Transition intensities and transition probabilities were estimated for movement among cognitive states, with corresponding 95% confidence intervals (CIs). We also estimated the expected time spent in each cognitive state during follow-up. Covariates were incorporated into the multistate model to evaluate their associations with specific cognitive transitions. Multistate Markov models have been used in longitudinal aging research to characterize both progression and reversibility across health states.(18)

## Results

### Study Population

Table 1 included the summary statistics of the study sample. Between 1992 and 2022, there were 10,489 participants with onset MCI, defined as a transition from previously observed normal cognition to MCI. At the index MCI wave, respondents had a mean age of 70.6 years (SD = 11.53), and 56.6% were female. About 72.4% of respondents were identified as White, 20.0% as Black or African American, and 7.4% as other races. More than half of participants were married or partnered (57.8%). Approximately one-quarter (25.9%) had less than a high school education, whereas 13.8% had completed college or higher education. At baseline, participants had a mean of 2.29 (SD, 1.51) chronic conditions. Medicare coverage was reported by 67.2%, and 9.1% reported no health insurance. Self-rated health was most commonly reported as good (32.9%), fair (26.7%), or very good (21.8%). Medicare coverage was reported by 67.2%, and 9.1% reported no health insurance. Self-rated health was most commonly reported as good (32.9%), fair (26.7%), or very good (21.8%).

The number of participants available for follow-up declined over time, from 6,952 in 2 years following the initial MCI to 4,240 in 6 years following the initial MCI onset and 2,380 in 10 years following the initial MCI onset. Respondents observed at later follow-up waves differed descriptively from the baseline cohort. For example, mean age was 70.6 years at baseline compared with 64.6 years among those observed at 10-year follow-up, and the proportion of married or partnered participants was 57.8% and 68.4%, respectively. Because these estimates describe the respondents remaining under observation at each wave rather than within-person changes, these differences should not be interpreted as longitudinal changes in individual characteristics.

### Cognitive Status Between Baseline and Observed Year

Substantial variation in cognitive status was observed following incident MCI (Fig. 1). At the 2-year follow-up, 62.3% of participants were classified as having normal cognition, 31.2% remained classified as MCI, and 6.5% were classified as having dementia.

With longer follow-up, the proportion classified as normal decreased, whereas the proportion classified as having dementia increased. At the 4-year follow-up, 57.5% were classified as normal cognition, 30.5% as MCI, and 11.9% as dementia; At the 6-year follow-up, the corresponding proportions were 51.2%, 34.8%, and 14.0%. These findings demonstrate substantial heterogeneity after incident MCI, including both reversion toward normal cognition and progression toward dementia.

### Transition intensity and transition probability

The continuous-time multistate Markov model demonstrated substantial bidirectional movement among cognitive states (Table 2). From MCI, the estimated transition intensity was 0.453 (95% CI, 0.443–0.463) for transition to normal cognition and 0.126 (95% CI, 0.121–0.131) for transition to dementia. Thus, the estimated instantaneous transition intensity from MCI to normal cognition was substantially greater than that from MCI to dementia.

Among participants classified as having normal cognition, the transition intensity was 0.265 (95% CI, 0.257–0.273) for transition to MCI and 0.037 (95% CI, 0.035–0.041) for transition to dementia. Transitions from dementia were also observed: the estimated intensity was 0.226 (95% CI, 0.209–0.245) for dementia to MCI and 0.064 (95% CI, 0.055–0.074) for dementia to normal cognition.

### Cognitive Transitions Between Successive Follow-up Waves

Transitions between adjacent observation waves further demonstrated the dynamic nature of cognitive status (Fig. 2). Between baseline and the 2-year follow-up, 62.3% of participants transitioned from MCI to normal cognition, 31.2% remained in MCI, and 6.5% transitioned to dementia. During subsequent adjacent intervals, MCI-to-normal transitions accounted for 9.7% to 13.4% of the participants observed at each respective wave, whereas MCI-to-dementia transitions accounted for 4.6% to 5.4%.

Recurrent transitions were also observed among participants who had previously moved to other cognitive states. Across the 2-to-4-, 4-to-6-, 6-to-8-, and 8-to-10-year intervals, 38.9% to 44.6% of the participants observed at each wave remained classified as normal, while 14.6% to 16.7% transitioned from normal cognition to MCI and 1.7% to 2.7% transitioned from normal cognition to dementia. Transitions from dementia to CIND accounted for 1.3% to 2.6% of participants observed at each respective wave, while transitions from dementia to normal cognition accounted for 0.5% to 0.8%.

## Discussion

In this population-based longitudinal study, cognitive trajectories following incident cognitive impairment were heterogeneous and dynamic. Rather than uniformly progressing toward dementia, participants experienced reversion to normal cognition, persistent cognitive impairment, and progression to dementia. Two years after incident MCI, 62.3% of participants were classified as having normal cognition, 31.2% remained classified as MCI, and 6.5% were classified as having dementia. Although dementia became more common with longer follow-up, substantial proportions of participants continued to be classified as normal or MCI. The multistate model further demonstrated bidirectional movement among cognitive states, with the transition intensity from MCI to normal cognition exceeding that from MCI to dementia. These findings support viewing cognitive impairment before dementia as a dynamic state characterized by competing possibilities of improvement, persistence, and deterioration, rather than solely as an intermediate stage in an inevitable progression toward dementia.

Our findings address an important gap in literature that has traditionally focused on progression from MCI to dementia. Increasing evidence indicates that MCI may remain stable or revert to normal cognition, particularly in population-based samples. A recent systematic review reported dementia conversion rates of 41.5% in clinical samples and 27.0% in population-based samples, whereas reversion occurred in 8.7% and 28.2%, respectively.(19) Our study extends this literature by examining multiple cognitive transitions simultaneously in a large population-based cohort. The substantial occurrence of MCI-to-normal transitions indicates that improvement and persistence are important components of prognosis after cognitive impairment and should be considered alongside progression to dementia. More broadly, factors associated with cognitive improvement may not simply be the inverse of those associated with deterioration, highlighting the importance of studying pathways toward cognitive resilience as well as dementia risk.

It is important to note that reversion to normal cognition did not necessarily mark the end of the cognitive trajectory. Respondents who returned to normal cognition could subsequently remain normal, transition back to MCI, or progress to dementia, demonstrating recurrent movement among cognitive states. Recent multistate research has similarly shown that individuals who revert from MCI to normal cognition may subsequently experience renewed cognitive deterioration. At the same time, longitudinal evidence suggests that individuals who revert may have more favorable subsequent cognitive and neurodegenerative trajectories than those with persistent MCI. Thus, reversion may not necessarily indicate permanent recovery. These findings illustrate an important limitation of many existing literature that classify MCI prognosis using only baseline status and a single follow-up outcome and support conceptualizing cognitive aging as a process involving recurrent transitions over time (20, 21).

Nevertheless, the high frequency of reversion observed in our study requires careful interpretation. Reversion in cognitive classification should not be equated with reversal of an underlying neurodegenerative process. Similarly, transitions from dementia to MCI or normal cognition should not be interpreted as dementia remission. Cognitive states in the HRS were determined using repeated cognitive assessments and classification thresholds rather than comprehensive clinical diagnoses. Consequently, observed transitions may reflect genuine changes in cognitive performance but may also reflect within-person variability, measurement error, testing circumstances, differences between self and proxy assessments, or movement around classification thresholds. Our findings therefore describe changes in cognitive classification rather than confirmed transitions among clinically diagnosed disease states. This distinction is especially important when interpreting less expected transitions from dementia to less impaired states.

These findings have important implications for clinical practice. An MCI classification should be viewed as an indicator of elevated cognitive vulnerability that warrants continued surveillance, rather than as a deterministic step toward dementia. Because cognitive status may improve, persist, or deteriorate over time, a single cognitive assessment may provide an incomplete picture of an individual’s longer-term trajectory. Repeated cognitive assessment may therefore help distinguish persistent impairment from transient or fluctuating changes and identify individuals experiencing renewed decline after apparent improvement. Moreover, reversion to normal cognition should not be interpreted as definitive recovery or as eliminating future risk; continued monitoring may remain appropriate even among individuals who subsequently test within the normal range. A longitudinal perspective that considers both current cognitive status and prior cognitive transitions may ultimately provide a more informative basis for clinical follow-up and patient counseling than cognitive classification at a single point in time.

Several limitations should be considered. First, cognitive states were based on TICS-derived and proxy-based research classifications rather than comprehensive clinical diagnoses. Observed transitions may therefore reflect both true cognitive change and classification variability. Second, cognitive status was generally assessed at approximately two-year intervals. Although the continuous-time multistate model accommodated interval-observed states, the exact timing of transitions was unknown, and multiple transitions occurring between assessments could not be directly observed. Third, classification using predefined score thresholds is susceptible to misclassification, particularly among participants with scores close to category cut points. Fourth, the number of participants with available cognitive assessments declined substantially over follow-up. Respondents observed at later waves differed from the baseline cohort, raising the possibility of selective survival and attrition and limiting interpretation of descriptive proportions at later follow-up. Finally, the absence of detailed neuropsychological assessments, clinical diagnoses, neuroimaging, and biomarkers prevented us from determining whether observed transitions corresponded to changes in underlying neuropathology. Future studies integrating these measures could help distinguish cognitive resilience from measurement-related fluctuation and clarify the mechanisms underlying different cognitive trajectories.

## Conclusions

In this population-based longitudinal study, cognitive impairment following incident MCI was characterized by substantial reversion, persistence, progression, and recurrent transitions among cognitive states. By considering these outcomes simultaneously, our findings extend the traditional progression-centered framework and support viewing cognitive impairment as a dynamic and bidirectional process. Reversion to normal cognition should not be interpreted as definitive biological recovery, but its frequent occurrence highlights an important dimension of cognitive aging that is obscured when dementia progression is considered as the primary outcome. Identifying factors that distinguish trajectories toward cognitive resilience, persistent impairment, and dementia may improve understanding of cognitive aging and help identify opportunities for intervention before dementia develops.

## Figures and Tables

**Figure 1:**
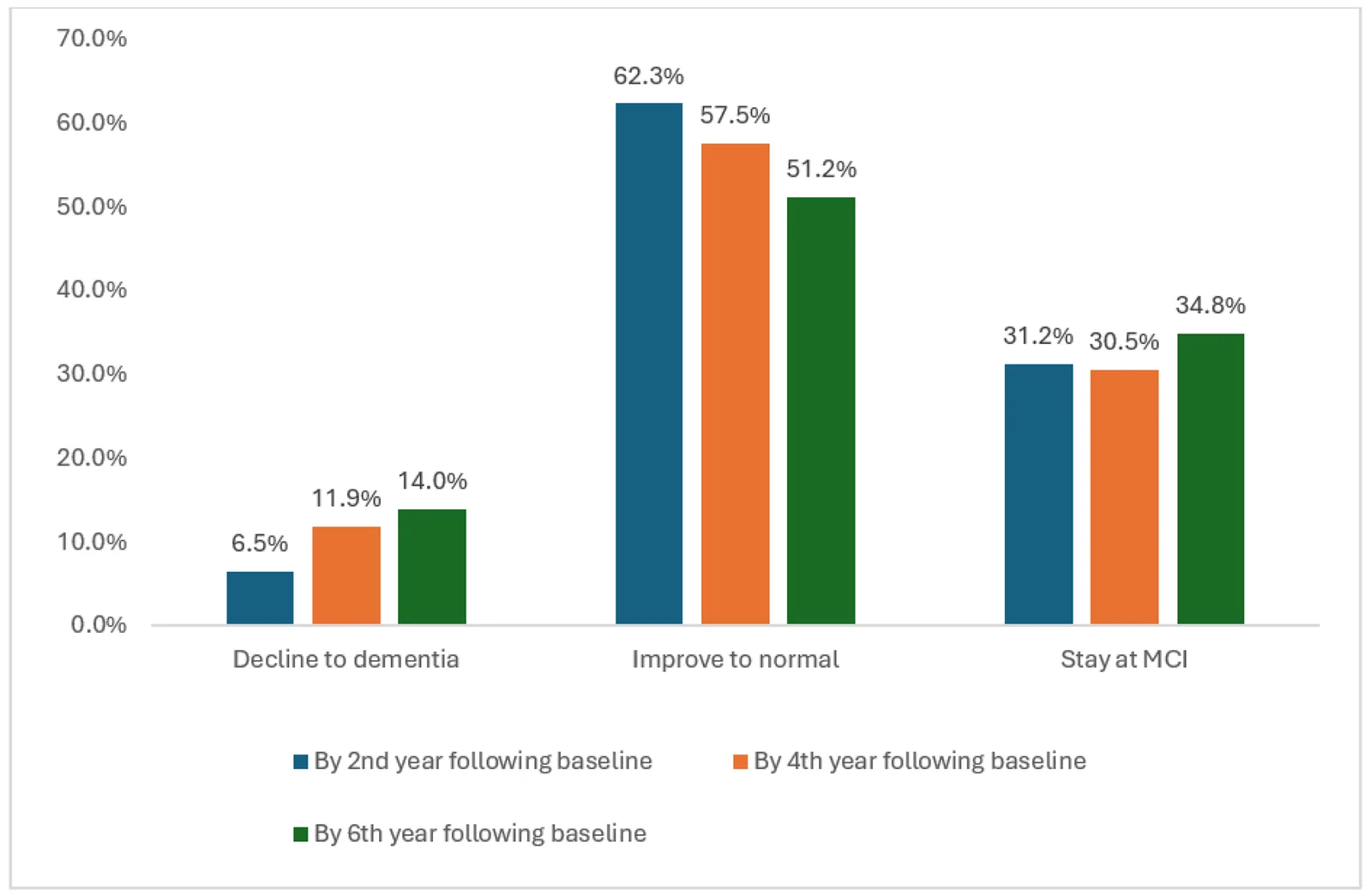
Change of Cognitive Status From Baseline to Follow up Survey Waves Among Respondents with Initial MCI at Baseline

**Figure 2:**
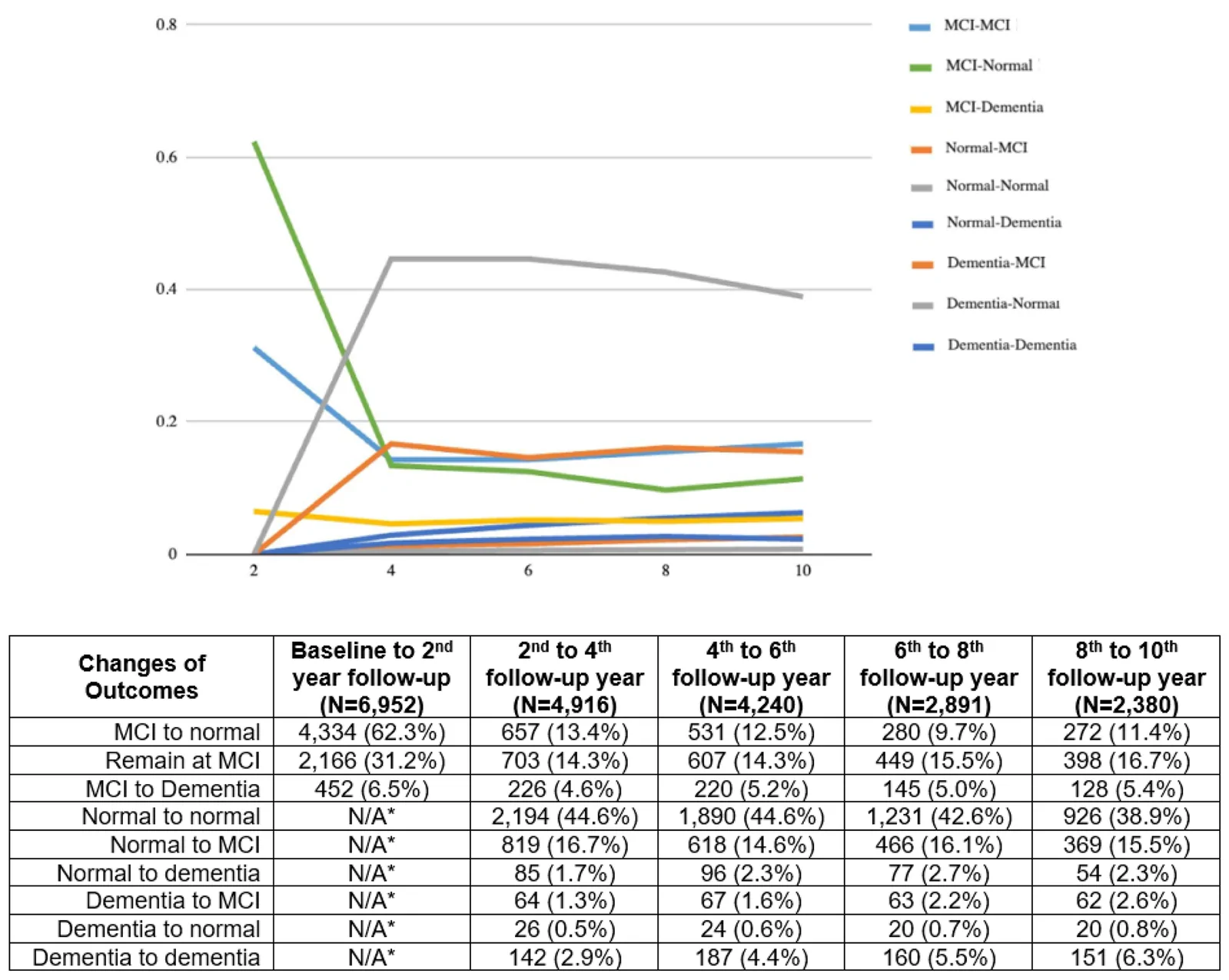
Estimated transition probabilities across 10 years. The graphical representation showcases the transition probabilities among states, derived from the estimated transition intensity, over a 10-year timeframe. The timeframes of years represent the time since the preceding state. *baseline to 2^nd^ year follow up: at baseline, all respondents had MCI. Therefore, only three possible changes occurred: MCI – normal, remain at MCI, and MCI to dementia.

**Table 1:** Summary Statistics of Study Population in Each Wave

Characteristics	Baseline/All Initial MCI (N = 10,489)	Two Years Following Initial MCI (N = 6,952)	Six Years Following Initial MCI (N = 4,240)	Ten Years Following Initial MCI (N = 2,380)
**Demographics**				
Age in years, mean (sd)	70.57 (11.53)	68.32 (10.89)	66.77 (9.86)	64.59 (8.83)
Age in groups				
< 65	3,696 (35.2%)	2,898 (41.7%)	1,942 (45.8%)	1,267 (53.2%)
65–74	2,647 (25.2%)	1,881 (27.1%)	1,283 (30.3%)	766 (32.2%)
75–84	2,763 (26.3%)	1,623 (23.3%)	837 (19.7%)	309 (13.0%)
>=85	1,383 (13.2%)	550 (7.9%)	178 (4.2%)	38 (1.6%)
Gender Sex				
Male	4,553 (43.4%)	3,168 (45.6%)	1,926 (45.4%)	1,080 (45.4%)
Female	5,936 (56.6%)	3,784 (54.4%)	2,314 (54.6%)	1,300 (54.6%)
Race				
White or Caucasian	7,595 (72.4%)	4,814 (69.2%)	2,869 (67.7%)	1,579 (66.3%)
Black or African American	2,103 (20.0%)	1,547 (22.3%)	1,016 (24.0%)	596 (25.0%)
Missing	14 (0.1%)	14 (0.2%)	9 (0.2%)	3 (0.1%)
Others	777 (7.4%)	577 (8.3%)	346 (8.2%)	202 (8.5%)
Ethnicity				
Not Hispanic	9,175 (87.5%)	5,958 (85.7%)	3,590 (84.7%)	1,990 (83.6%)
Hispanic	1,308 (12.5%)	989 (14.2%)	648 (15.3%)	389 (16.3%)
Missing	6 (0.1%)	5 (0.1%)	2 (0.0%)	1 (0.0%)
Marital Status				
Married/partnered	6,069 (57.8%)	4,651 (66.9%)	2,870 (67.7%)	1,627 (68.4%)
Separated/divorced/widowed	1,429 (13.6%)	1,064 (15.2%)	656 (15.4%)	360 (15.2%)
Never married or missing	2,991 (28.5%)	1,237 (17.8%)	714 (16.8%)	393 (16.4%)
Education				
Less than high school	2,720 (25.9%)	1,886 (27.1%)	1,259 (29.7%)	756 (31.8%)
High school	4,052 (38.6%)	2,679 (38.5%)	1,624 (38.3%)	936 (39.3%)
Some college	2,263 (21.6%)	1,463 (21.0%)	850 (20.0%)	446 (18.7%)
College or above	1,452 (13.8%)	924 (13.3%)	507 (12.0%)	241 (10.1%)
Missing	2 (0.0%)	0 (0.0%)	0 (0.0%)	1 (0.0%)
**Health insurance**				
Medicare	7,047 (67.2%)	4,260 (61.3%)	2,411 (56.9%)	1,185 (49.8%)
Medicaid	1,176 (11.2%)	815 (11.7%)	462 (10.9%)	249 (10.5%)
CHAMPVA / TRICARD	581 (5.5%)	372 (5.4%)	243 (5.7%)	116 (4.9%)
Employer sponsored	4,915 (46.9%)	3,349 (48.2%)	2,137 (50.4%)	1,272 (53.4%)
No insurance	955 (9.1%)	730 (10.5%)	492 (11.6%)	316 (13.3%)
**Medical conditions**				
# chronic conditions	2.29 (1.51)	2.09 (1.48)	1.92 (1.41)	1.70 (1.32)
**Self-reported health status at MCI**				
Excellent	710 (6.8%)	508 (7.3%)	336 (7.9%)	225 (9.5%)
Very good	2,284 (21.8%)	1,544 (22.2%)	988 (23.3%)	563 (23.7%)
Good	3,454 (32.9%)	2,366 (34.0%)	1,429 (33.7%)	795 (33.4%)
Fair	2,798 (26.7%)	1,795 (25.8%)	1,084 (25.6%)	212 (8.9%)
Poor/missing	1,243 (11.8%)	739 (10.6%)	403 (9.5%)	215 (9.0%)

**Table 2:** Transition intensities of Multistate Markov model

MCI	MCI	Normal	Dementia
	
	−0.579* **(−0.592, −0.565)**	**0.453** **(0.443, 0.463)**	**0.126** **(0.121, 0.131)**

Normal	0.265 (0.257, 0.273)	−0.302* **(−0.311, −0.293)**	0.037 (0.035, 0.041)

Dementia	0.226 (0.209, 0.245)	0.064 (0.055, 0.074)	−0.290* **(−0.312, −0.269)**

Notes: The negative values indicate the outflow of the patients from MCI, normal or dementia. The value of outflow from MCI (−0.579) equals the value of inflow of from MCI to normal and MCI to dementia (0.453 + 0.126). The value of outflow from normal (−0.302) equals the value of inflow from normal to MCI and normal to dementia (0.265 + 0.037). The value of outflow from dementia (−0.290) equals the value of inflow from dementia to MCI and dementia to normal (0.226 + 0.064).

## Data Availability

The study is a secondary data analysis and do not generate any data. The data used in this study are from the Health and Retirement Study (HRS), a nationally representative longitudinal study of adults aged 50 years and older in the United States. HRS data are available through the University of Michigan’s HRS Data Portal at https://hrsdata.isr.umich.edu/, following user registration and subject to applicable data use conditions.

## Abbreviations

MCI: Mild cognitive impairment
